# Experiences and needs of the caregivers of stroke survivors in Malaysia—A phenomenological exploration

**DOI:** 10.3389/fneur.2022.996620

**Published:** 2022-09-23

**Authors:** Norsima Nazifah Sidek, Sureshkumar Kamalakannan, Tengku Alina Tengku Ismail, Kamarul Imran Musa, Khairul Azmi Ibrahim, Zariah Abdul Aziz, Iliatha Papachristou Nadal

**Affiliations:** ^1^Department of Community Medicine, School of Medical Sciences, Universiti Sains Malaysia, Kubang Kerian, Kelantan, Malaysia; ^2^Clinical Research Centre, Hospital Sultanah Nur Zahirah, Kuala Lumpur, Terengganu, Malaysia; ^3^Department of Social Work Education and Community wellbeing – Northumbria University, London, United Kingdom; ^4^Department of Non-communicable Disease and Epidemiology, London School of Hygiene and Tropical Medicine, London, United Kingdom; ^5^Department of Medicine, Hospital Sultanah Nur Zahirah, Kuala Lumpur, Terengganu, Malaysia

**Keywords:** stroke survivor, road to recovery, informal caregiver, caregiving, caregiver needs, stroke care

## Abstract

**Introduction:**

Informal caregivers play a crucial role in supporting the activities of daily living, rehabilitation, and the road to recovery for stroke survivors. However, these informal caregivers are often reported as experiencing neglect and lack of recognition despite their primary contribution to stroke recovery. This study investigated the experiences of the caregivers of stroke survivors and access to stroke care in Malaysia.

**Method:**

This qualitative study with a phenomenological approach utilized in-depth interviews, including ten primary caregivers, one formal caregiver, and stroke healthcare providers as the participants. The interviews were done until the data saturation was achieved, and the data was analyzed using thematic analysis.

**Result:**

Three primary themes and 14 subthemes were identified from the interviews. The role of primary caregivers of stroke survivors had tremendous physical, mental and social impact on the caregivers. Caregivers had two primary needs. The need for information about comprehensive stroke care at home and the need for psychological support to themselves. The key internal driver for providing care was identified to be the motivation level of the stroke survivor and the external driver was identified to be the societal support with access to comprehensive stroke care.

**Conclusion:**

The role of informal caregivers becomes critical for continuum of stroke care. As caregivers take up the roles and responsibilities of those who contribute to stroke rehabilitation single-handedly soon after hospital discharge. Results of this study highlights the needs for providing systematic support to caregivers for engaging them in effective stroke care, particularly in the community. Stroke service providers, policy makers and program planners must be sensitized to empower caregivers of stroke survivors in effectively supporting stroke survivor in their family on the road of recovery.

## Introduction

For the past three decades, stroke has been the second leading cause of death and the third leading cause of disability worldwide (1). This situation is not so different in Malaysia. Stroke ranks as the third leading cause of death in Malaysia, accounting for about 7.8% of all deaths in the past 5 years (2). Due to advancements in medical care, approximately 90% of stroke victims survive their initial stroke event (3). However, about 50% develop stroke-related impairments that limit their independence in everyday activities and functioning (3, 4). This implies the substantial need for rehabilitation and support required by stroke survivors, especially outside the hospital setting (5). These support needs are met by the primary caregivers, usually the stroke survivors' family members. The caregiver's level of support depends on the severity of the disability following the stroke (6).

Due to cultural norms pertaining to family obligation, the family usually assumes the role of caregiver in Malaysia (7). These informal caregivers assumes the responsibility to support the stroke survivors in their daily living activities, including care and support for their therapy and rehabilitation needs (8). Although feasible, the cost for post-stroke outpatient care was substantial for the stroke survivors ranging from USD 53.50 to USD 4,591.60 depending on stroke severity (9). This reflects the unmet need for stroke care in the community.

With the increasing number of poor households and the soaring levels of absolute poverty in Malaysia, financial constraints may become an additional burden for caring for the stroke survivor (10). It is also essential to recognize the opportunity cost and the community-level barriers such as limited public support and community-based rehabilitation services for addressing the growing needs of the stroke survivors and the support to their caregivers at home (11). This situation increases the strain and growing burden on caregivers to support their family members affected by stroke-related disability (7, 8, 11–13).

Although there is quantitative evidence for the stress, strain and burden experienced by the caregivers of stroke survivors, an in-depth understanding of the context-specific experiences and perceptions of the caregivers of stroke survivors in Malaysia is not known (14). In general, they experience neglect and lack of recognition in the family for all their efforts and sacrifices for being a primary caregiver (12). Nevertheless, their well-being is as important as the stroke survivors since they play a crucial role in the rehabilitation and the road to recovery for stroke survivors (15, 16).

Therefore, an in-depth understanding of the caregiver's experiences in looking after a stroke survivor to improve the care of stroke survivor and effectively empower them in the community is warranted (17). Hence, this study investigated the experiences of caregiving for stroke survivors and access to stroke care in Malaysia. This study is a part of a larger project aimed at developing a mobile health application for caregivers of stroke survivors in Malaysia. The findings from this study are expected to inform the development of a client-centered, culturally relevant solution for the caregivers of stroke survivors in Malaysia.

### Objectives

- To understand the experiences and needs of family caregivers in providing care for the stroke survivors in their families based on the caregiver themselves.
- To understand the experiences of access to stroke care services from the perspectives of caregivers (CG) and rehabilitation service providers (HCP).

## Materials and methods

### Study design

This is a qualitative study with a phenomenological approach. In-depth interviews with the participants were the primary method of data collection.

#### Participants

**a. Caregivers of stroke survivors (CG):**
• Caregivers of stroke survivors were selected from a list of patients who had attended rehabilitation and neurology clinics at the three hospitals in Terengganu (Hospital Sultanah Nur Zahirah) and Kelantan (Hospital Raja Perempuan Zainab 2 and Hospital Universiti Sains Malaysia).
• Caregivers had to support a stroke survivor who had a confirmed diagnosis of stroke.
• The stroke survivor for whom care was provided must be functionally dependent (Modified Rankin Scale 4 or 5).
• The caregiver must be an adult aged 18 years and above and had been the primary caregiver for at least 3 months.
• Willing to participate in research, be interviewed, and share their information and experiences.

**b. Healthcare Providers (HCP)**
• A list of healthcare providers, including neurologist, rehabilitation physicians, rehabilitation nurses, occupational therapists, physiotherapists, and formal caregivers, was provided by the stroke team representatives at the three hospitals in Terengganu (Hospital Sultanah Nur Zahirah) and Kelantan (Hospital Raja Perempuan Zainab 2 and Hospital Universiti Sains Malaysia).
• Healthcare providers were eligible only if they were actively involved in the care of stroke survivors for at least a year and were willing to participate in the study.

#### Sampling

Twelve healthcare providers and ten primary caregivers of stroke survivors were included. We intended to explore the experiences of various specialists involved in stroke rehabilitation; hence healthcare providers were chosen from different professions and service settings. Similarly, we planned to obtain diverse backgrounds from caregivers; therefore, we included caregivers with varying age groups and genders (for caregiving and care receiving), relationships (spouse, children, sibling), education level and duration of caregiving (18–20).

The selection of potential caregivers was based on purposive sampling techniques by reviewing the stroke survivors' medical records to obtain information on their Modified Rankin Scale (MRS) scores, which were used to determine their dependency status and identify their caregiver as well as the caregiver contact number. As for the healthcare provider, we use the snowball convenience sampling method to select the potential participants. The main person involved in stroke rehabilitation was contacted and given a brief study overview. Then we asked for the names of people who might be interested in participating in the interview. The potential participants eligible for the study were contacted by phone.

During the initial contact, the purpose of the study and what participation entailed were explained to potential participants. If they expressed an interest in participating, the study information and electronic informed consent were sent to them. Once the consent was obtained, the interview dates and times were scheduled based on their availability and preference.

### Investigation of the experiences

In-depth interviews were used as the primary method to investigate the experiences of participants related to caregiving and access to stroke care. Specific topic guides were developed for the purpose of the interviews and were piloted to refine the topic guides to cover the experiences of participants comprehensively (Table 1). The interviews were conducted between June and November 2021. The original plan was to conduct the interviews in person. However, because of the COVID-19 pandemic, associated control measures, and physical distance constraints, the interviews were performed over the phone or through the Webex@ platform, whichever was most convenient for the participants. This strategy is also intended to make participants more comfortable, allowing them to divulge sensitive information due to greater discretion while bridging the geographical gap (21, 22). The interviews were conducted by one of the investigator, in the interviewees' preferred language (Malay or English) and were recorded.

**Table 1:** Topic guide for in-depth interviews.

**Topic**	**Main**	**Probe**
**For healthcare provider**		
Post-stroke care service	Can you explain about the post-stroke care Service available here?	The context for the provision of post-stroke care services Barriers Facilitators Managing barriers and optimizing facilitators
Quality of service	What do you think about the current post-stroke care service?	Perceptions and opinions Benefits Drawbacks
Caregiving of stroke survivor	Based on your experience in managing post-stroke patients and meeting the caregiver, what do you think about the caregiving of stroke survivors here?	Perception and opinion Barriers Facilitators Needs Support provided for caregiver
**For caregiver**		
Post-stroke care service	Can you share your experience in accessing the post-stroke care service?	Context of receiving the post-stroke care services Quality of the service Barriers Facilitators Managing barriers and optimizing facilitators
Quality of service	What do you think about the current post-stroke care service offered?	Perceptions and opinions Benefits Drawbacks
Caregiving of stroke survivor	Can you share your experience in the caregiving of stroke survivors?	Barriers Facilitators Needs Support received for caregiving

### Data saturation

In total, 22 participants (12 healthcare providers, and ten caregivers) were recruited for the study, as this was the point at which the interviews reached saturation. After the 20th interview, there was no new information generated from the interviews. Therefore, it was deemed that the data collection had reached a saturation point. We continued the data collection by conducting two more interviews to ensure and confirm that there was no new information that emerged from the interviews.

### Ethical consideration

The study was approved by the Malaysia Research Ethics Committee (MREC Ref: KKM/NIHSEC/P20-922), The Human Research Ethics Committee of *USM* (*JEPeM*): USM/JEPeM/20010031, and the *London School of Hygiene and Tropical Medicine* (*LSHTM)* Research *Ethics Committee (*LSHTM Ethics Ref: 19079). The participants were informed both verbally and in writing about the aim of the study, the voluntary participation, and the confidential handling of their audio recordings. All interview transcripts were anonymized immediately after data collection. Participants were offered an honorarium (RM 50 for the healthcare provider and RM100 for the caregiver) to compensate for their time.

### Data analysis

Data were managed using NVivo 1.6.1 (released in January 2022). Data analysis was guided by Braun and Clarke's six-phase process of thematic analysis (1. familiarize yourself with the data, 2. generate initial codes, 3. searching for themes, 4. reviewing themes, 5. defining and naming themes, 6. producing the report) (23). In phase 1, all interview audio recordings were downloaded and transcribed verbatim in original language. To ensure consistency and rigor in translation, the interviews conducted in Malay were translated into English by two bilingual research investigators to ensure reliability of the translated information. This was done by switching back and forth between the raw data and the translated data which were subsequently double-checked and refined by the research team (24). In order to ensure privacy and data management, the data from each participant was assigned identifiable codes.

### Evaluation and trustworthiness

To ensure trustworthiness, this study was based on Lincoln and Guba Four-Dimension criteria (FDC): credibility, confirmability, dependability and transferability (25). For validation purposes, member-checking was done by sharing the results with a few participants to ensure that they accurately reflected their perceptions, feelings, and experiences shared during the interviews. The researchers also used investigator triangulation by checking preliminary findings and interpretations against the raw data to reduce researcher bias. To establish credibility, the researcher went through constant analysis and allocated sufficient time for data collection and more prolonged involvement in the research to obtain a thick and rich data set for confirmability. To achieve the criteria of transferability, all details of the research were described, from sampling to data collection and analysis. For the reliability of the study, we continually compared the data with the codes, prepared a codebook with the description or definition for each code, and conducted regular meetings with the research group to confirm coding and categorizing processes. These multiple approaches were used to enhance and ensure the data analysis accuracy and study findings (26).

## Results

The demographic characteristics of the 22 participants who were interviewed (ten caregivers and 12 healthcare providers) are provided in Table 2. The interviews lasted an average of 55.5 min (SD 14.84). The caregiver group of participants were diverse in terms of their age, years of experience in caregiving, gender, education and employment status. The stroke survivors they cared for were all above 50 years of age. Similarly, the healthcare providers group interviewed were also diverse, especially regarding their professional expertise and years of providing services for stroke survivors.

**Table 2:** Participants' characteristics.

**Characteristic**	**Healthcare** **providers (*n* = 12)**	**Informal caregiver** **(*n* = 10)**
**Age (years)**		
21–30	4	3
31–40	4	1
41–50	3	1
51–60	1	3
61–70	0	2
**Gender**		
Male	4	5
Female	8	5
**Relationship to patient**		
Spouse	–	4
Son/daughter	–	5
Sibling	–	1
**Duration of caregiving**		
< 6 months	–	2
6–12 months	–	2
1–5 years	–	4
>5 years	–	2
**Education level**		
Primary	–	0
Secondary	–	5
Tertiary	–	5
**Work status**		
Employed/self employed	–	3
Unemployed/retired	–	7
**Stroke survivor gender**		
Male	–	5
Female	–	5
**Stroke survivor age (years)**		
51–60	–	4
61–70	–	5
>70	–	1
**Professional background**		
Neurologist/rehab physician	2	–
Physiotherapist		–
Occupational therapist	3	–
Nurse	3	–
Homecare manager	3	–
**Stroke management experience**		
1–5 years	6	–
>5 years	6	–

### Themes and sub-themes

**Figure 1:**
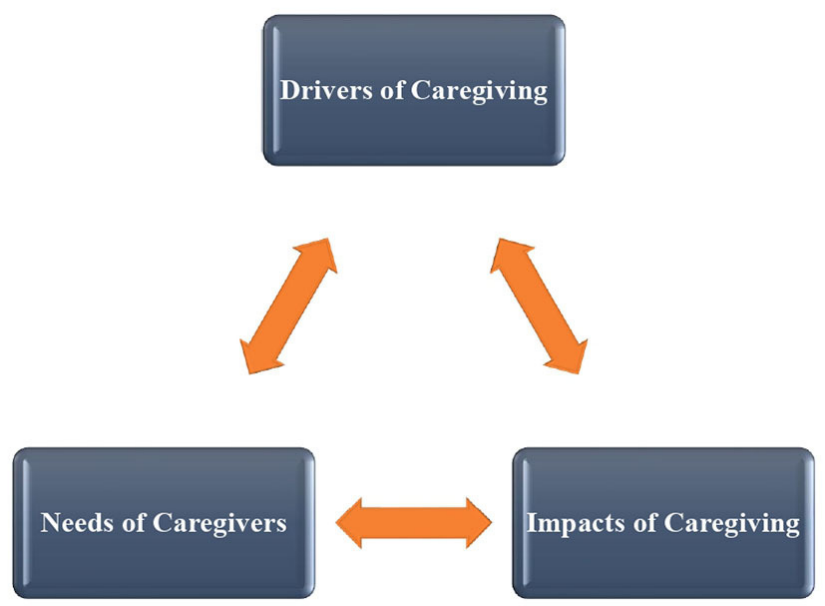
Relationships of the themes identified in the study.

Three primary themes and 14 subthemes were identified from the interviews (Table 3). The three primary themes were a. Impact of the caregiving experience on caregivers b. Needs of the caregivers c. Internal and external drivers for caregiving. The important quotes related to each theme are provided, and other examples are available in Supplementary Table 1. The pictorial representation of their relationships derived from the study is depicted in Figure 1.

**Table 3:** Summary of themes and subthemes.

**Themes**	**Subthemes**
Impact of caregiving	• Impact on the mental and emotional well-being of the caregivers • Impact on the physical health of the caregivers • Impact on the social and professional life of the caregivers
Needs of the caregivers	• Educational and informational support to caregivers • Psychological support requirements and needs of caregivers
Drivers for caregiving (internal and external)	• Key characteristics of caregiver • Factors within stroke survivor themselves • Family and societal support • Restriction due to pandemic • Sub-optimal quality for formal stroke care • Healthcare provider emotional support • Healthcare provider informational and instrumental support • Support for essential

#### Impact of the caregiving experience on the caregivers

The role of a primary caregiver of a stroke survivor had a tremendous mental, physical and social impact on the personal as well as professional lives of the caregivers. Everyday routines, lifestyle and family situations were drastically altered negatively. It increased the workload in relation to the caregivers' roles and responsibilities, mainly because they were expected to assume an additional role in the family and take over all the duties of the stroke survivor in their family. Given this sudden change in the family situation, and without any prior experience of this life-changing situation, the caregivers generally felt unprepared, overwhelmed and burdened to manage the family situation.

The caregiver experienced psychological distress and exhaustion due to caregiving. Primarily because of their additional responsibilities and change of routine due to the sudden stroke event in the family. The other reason mentioned was that they were unaware of the appropriate way to support a stroke survivor at home. They felt depressed and emotionally distressed. The health care providers even expressed this state of mental status. The caregivers also mentioned about the role of caregivers and its impact on their physical health. Providing care for the stroke survivor was physically draining them and as the health of the family member surviving a stroke cannot be compromised, it was essential for the caregivers to support the stroke survivor in their routine tasks such as assisting them for toileting, washing them, feeding them and sometimes positioning them in bed or chair. They commonly reported sleep deprivation. Some caregivers reported that they almost neglect their own medical issues because they focus on supporting the stroke survivor in their family.

The majority of the caregivers interviewed expressed that they had sacrificed their own needs in order to assume their new role as a caregiver and support the stroke survivor in their family. Some caregivers explained that the responsibilities were too much that they had to quit their job in order to fully take up the responsibility of caregiving and supporting the stroke survivor. Some caregivers are required to take time away from their jobs to care for stroke survivors. As a result, they reported that their job performance and the quality of their work has suffered as a direct result of this obligation.

“*The father took care of the mom (patient) until he had a low blood pressure. When we called to see the patient, he said, I warded, because I have a low blood pressure, warded because of hypotension” (HCP 2; Domiciliary nurse)*

“*Ha, of course I am stressed. With my own child having a tantrum, PdPR (Home-based teaching and learning) some more, because everything is on us, ha..cooking.. rushing…always rushing, keep looking at the clock. Always had to plan, oh after this, what should I do, then what….” (CG 4)*

#### Needs of the caregivers

Under this theme, the caregiver has highlighted some of the needs they felt essential to ease their individual, family, and social roles in addition to their role as a caregiver. There were two kinds of needs expressed. The first kind of need is related to information on stroke and stroke rehabilitation. Caregivers indicated their need to be aware of stroke, complications and disability caused by stroke, and the approaches to managing disabilities following a stroke at home. Stroke education was their topmost need. Both caregivers and healthcare providers mentioned the importance of gaining awareness and knowledge about the stroke impact, rehabilitation, and prevention when a caregiver supports a stroke survivor. This is mainly to help them encourage the stroke survivors to become motivated toward recovery process without any recurrence and complications due to their stroke in the future. They believed that caregiving could become much easier if the patient is emotionally stable.

The second was psychological support. The caregivers expressed that they need this support to mentally equip them to support the caregivers without being emotionally distressed. This was to meet the demanding needs of the stroke survivors and strike an effective balance between their individual, family, and social roles and responsibilities. Healthcare providers also recognized caregiving's psychological impact and recommended timely mental health assessment and psychological support by trained counselors to the caregivers of stroke survivors.

“*Yes. Counselling for the caregiver. So that we can share our feeling, we can express our feeling, at least there is someone who listen to us. Hmm. Because I think, sometimes, people got really depressed, and they cannot handle it and end up accidently kill…., that kind…Because they felt, oh this patient is a burden to them. It's would be better if they gone.haa..it's like that. Who knows this thing…” (CG 7)*

“*So that is why when I say when we do this outcome measures, DASS, for example, we actually need to engage the counselling site because counselling will help to make the client caretaker to understand the situation, and as we know, stroke is a disease that is lifelong and we cannot simply say, “okay today you do this, tomorrow you are going to walk”. (HCP 5; Physiotherapist)*

#### Key drivers for caregiving

This study identified important factors that could drive effective caregiving for stroke survivors. The drivers were of two kinds. One is internal reflecting the factors that enable effective caregiving within the family context, including the stroke survivor, their caregiver, and the other family members. The other one is external, reflecting the factors that enable effective caregiving, including the health and social care systems and the rehabilitation context.

#### Internal drivers (within the family involved)

The first and foremost factor that drives caregiving is the stroke survivor themselves in the family. A stroke survivor with good motivation was frequently described as an essential driver for rehabilitation and caregiving. Both caregivers and healthcare providers mentioned that stroke survivors who exhibiting apathy, anhedonia, and de-motivation were extremely taxing for the caregivers to support. Stroke survivors with such motivation levels were considered to recover more slowly, and the efforts from the caregivers were non-productive. The second important driver for caregiving is the caregiver. Importantly their willingness and commitment to providing care and their attitude toward the stroke survivors. The healthcare providers considered lack of motivation and commitment by the caregiver as potentially harmful for the stroke survivor. They believed that a positive mindset and a strong commitment were necessary for effective caregiving that could aid in rehabilitation. The third but most important driver is the economic situation of the family. Caregivers expressed that if the stroke survivor was the only earning member of the family, there would be devastating consequences for the entire family, let alone providing care and support. The caregivers expressed that this situation is especially true soon after the stroke survivor is discharged from the hospital and sent home. Acknowledged by the healthcare providers, given the lack of community-based rehabilitation services, the cost of care was considered to place humongous financial stress on the entire family. As a result, they felt the care and support to the stroke survivor become compromised and ineffective as the financial situation places an additional burden on the family.

“*He refuses to walk, He can, the therapy people said, he can. Can walk, uh walk on his own with the cane, the single stick cane. But he didn't. Like he's not confident..ha..he himself don't feel confident to walk.. to walk on his own, because he afraid he will fall (CG9)*

“*From the financial part, sometimes we ask them to buy..like ripple mattress..uh..the better one…em..then we ask to buy the walking frame, um..for..uh..to practice..Then we ask to buy..like patient just lying on the floor, so we ask to buy a proper bed..or we can use any available bed in their home for instance..Ha.. so if the caregiver with poor financial, he has no money, he couldn't buy all these things.” (HCP6; Occupational Therapist)*

#### External drivers (outside the family)

The first and foremost driver outside the family for effective caregiving reported by both caregivers and healthcare providers is societal support. The caregivers expressed their desperation to find another caregiver and share or seek solutions for effective caregiving. On top of that, most healthcare providers and caregivers expressed the lack of accessibility and availability of rehabilitation services in general in the country. Lack of effective rehabilitation systems, including inadequate rehabilitation workforce, infrastructure, and affordable assistive technology supplies, were reported as key reasons for poor prognosis and ineffective management of stroke survivors in the community.

The participants recommended for community-based rehabilitation services that enable the stroke survivor and the caregiver to self-equip themselves with effective stroke recovery that is affordable and available for every stroke survivor in Malaysia, irrespective of their financial situation, to access such services. The participants, especially the healthcare providers, also recommended the development of effective pathways for follow-up and coordinated care of stroke survivors discharged from the hospitals with community-based solutions for adequate provision of care. Caregivers expressed that it would be immensely helpful if these services with appropriate training and support could guide them right from the first day of acute stroke treatment. Even the healthcare providers suggested a system to support the caregivers in providing effective care at home. Lastly, with the pandemic situation, participants expressed that even the existing services were deemed inaccessible and had impacted negatively on the recovery of the stroke survivor. This subsequently made the caregivers more vulnerable to the risk of physical, mental, and emotional distress leading to untoward consequences at all levels.

*I think it is the same. I think, in in Malaysia the post hospital care is not well developed in our country. (HCP 10; Neurologist)*

*For the bedridden patient, meaning like this domiciliary patient, they are a bit affected because we cannot go…. because lack of staff, sometimes our staff go to NCD [non-communicable disease] team, our staff at PPV [vaccination centre], so it's really shortage staff for domiciliary care nowadays (HCP 2; Domiciliary nurse)*

## Discussion

The experiences and needs of the caregivers of stroke survivors in Malaysia clearly demonstrated the appalling situation of those suffering from stroke and their family and, more importantly, the vulnerability and negative consequences for the caregivers. To our knowledge, this is the first study to explore the experiences of caregiving and access to stroke care inclusively with caregivers and healthcare providers in Malaysia. It was evident from the themes and sub-themes that the experiences of providing informal care for dependent stroke survivors at home are multidimensional, resulting in devastating family situations and poor quality of life for those in the entire family, particularly among the primary caregivers of the stroke survivors.

Numerous drivers influence caregiving. The support from the caregiver was identified as the main driver for successful caregiving. This support was determined to be related to their level of understanding toward stroke, the approach to stroke care, trust and attitude toward caregiving, and the family's financial situation. These findings were very similar to recent global studies on stroke and other conditions such as dementia, elderly care, and schizophrenia (27–30). In line with the existing literature, depression and emotional disturbances in stroke survivors are known to be the frequent problems that negatively impact the provision of care and recovery (31, 32). Similar to other study findings, societal support, as well as the support from healthcare providers was found to play a crucial role in the success of caregiving (33–35). However, due to the sub-optimal level of access to stroke care, not all caregivers and stroke survivors receive the available benefits. Findings from this study highlight the lack of access to stroke care, especially outside the hospital settings, and the increasing demand for informational and psychological support to the caregivers of stroke survivors in the community. The failure to meet these needs of caregivers with scalable solutions could potentially trigger devastating consequences on their emotional, physical, social, and professional lives.

The unpreparedness for the sudden assumption of a new role, additional responsibilities, abrupt change in family dynamics, and a change in their life expectations were the important factors that explained the negative experiences of the caregivers. These findings were supported by previous literature too (15, 16, 19, 36, 37). Even the healthcare providers were able to express their awareness of this situation in the interviews and recommended the need for appropriate psychological support and training to the caregivers of stroke survivors right from the beginning of their journey as a caregiver. This approach has been shown to reduce psychological distress, caregiver burden, and strain effectively (38–40). However, in the context of Malaysia, access to such services is still not foreseeable.

Most caregivers lack awareness and knowledge about stroke care and proper caregiving techniques, resulting in them focusing solely on the nursing aspect rather than managing disabilities following stroke and rehabilitation (19, 41–43). Interventions to empower the caregivers of stroke survivors could improve the quality of stroke care and enhance the recovery of the stroke survivor. This strategy has been shown to improve caregiving skills and competencies and reduce caregivers' stress and burden (20, 44).

Given the inaccessible context for stroke services, the interventions for caregivers require innovative approaches such as the use of technology and advances in remote management of health (45, 46). Mobile health apps that are systematically designed and developed have proven feasible and acceptable for supporting the management of disability in the community for stroke survivors and caregivers (47–50). This could be amalgamated with the ongoing health system strengthening efforts and response to the pandemic to both support the health systems as well as address the growing needs of stroke survivors and their caregivers in Malaysia. The lack of system support in providing care for stroke survivors and its effect was well demonstrated even in high-income countries (41). It is of immense public health importance to systematically develop scalable solution such as a mobile health application for stroke survivors and their caregivers in Malaysia to address the unmet need for rehabilitation and to prevent those in need from additional vulnerabilities and their consequences (51). There is a massive implication for this innovative solution not just for stroke care but also for the current pandemic situation and not just in Malaysia but worldwide (51).

There were two limitations of this study. Firstly, lack of stratification of participants based on ethnicity as the majority of the participants was Malay. Secondly, regarding the geography factor, the participants were recruited from two states of East Coast Malaysia. The stroke rehabilitation care and service may differ in other states, especially in the central region. Hence, further studies in the future could involve more states with ethnic and socioeconomic diversity.

Despite the limitation, this study provides valuable and deeper insights into effectively organizing stroke care, rehabilitation, and disability management in Malaysia. Scalable solutions are highly pertinent to empowering the caregivers of persons with disabilities, especially in Malaysia's pandemic and similar contexts globally.

## Conclusion

Caregivers are an essential part of the lives of a person with disabilities in general. The role of informal caregivers becomes even more critical in a context where there are issues with access to rehabilitation services. Stroke has been the leading cause of disability worldwide for the past three decades contributing significantly to the growing global burden of disability. However, in a context like Malaysia, what is available for them to support stroke survivors are limited to those first few days of care at the hospitals.

The role of informal caregivers becomes critical for continuum of stroke care. As caregivers take up the roles and responsibilities of those who contribute to stroke rehabilitation single-handedly soon after hospital discharge. Results of this study highlights the needs for providing systematic support to caregivers for engaging them in effective stroke care, particularly in the community. Stroke service providers, policy makers and program planners must be sensitized to empower caregivers of stroke survivors in effectively supporting stroke survivor in their family on the road of recovery.

## Data availability statement

The raw data supporting the conclusions of this article will be made available by the author, without undue reservation.

## Ethics statement

The studies involving human participants were reviewed and approved by the Malaysia Research Ethics Committee (MREC Ref: KKM/NIHSEC/P20-922), the Human Research Ethics Committee of USM (JEPeM): USM/JEPeM/20010031, and the London School of Hygiene and Tropical Medicine (LSHTM) Research Ethics Committee (LSHTM Ethics Ref: 19079). The patients/participants provided their written informed consent to participate in this study.

## Author contributions

KM and SK supervised the project. NS, SK, TT, and IP designed the study and acquired and analyzed the data. NS, SK, TT, IP, and KM interpreted the results. NS and SK drafted the manuscript. TT, IP, KM, KI, and ZA made critical revision of the manuscript. All authors contributed to the article and approved the submitted version.

## Funding

This study was supported by the matching grant, Newton-Ungku Omar Fund from the Ministry of Higher Education, Malaysia (University Sains Malaysia 203.PPSP.6780003) and Medical Research Council, UK (London School of Hygiene and Tropical Medicine MR/T018968/1). The funders had no role in study design, data collection and analysis, preparation of the manuscript, or decision to publish.

## Conflict of interest

The authors declare that the research was conducted in the absence of any commercial or financial relationships that could be construed as a potential conflict of interest.

## Publisher's note

All claims expressed in this article are solely those of the authors and do not necessarily represent those of their affiliated organizations, or those of the publisher, the editors and the reviewers. Any product that may be evaluated in this article, or claim that may be made by its manufacturer, is not guaranteed or endorsed by the publisher.
