# Supplementary material for: Experiences and needs of the caregivers of stroke survivors in Malaysia—A phenomenological exploration
Source: Front Neurol. 2022 Sep 23;13:996620. doi: 10.3389/fneur.2022.996620 (PMC9539245; doi:10.3389/fneur.2022.996620)
Supplement: Supplementary file 1 [file Table_1.DOCX]

Supplementary Material

# Supplementary Tables 1: Example of Quote

| *Theme* | *Subtheme* | *Quote* |
| --- | --- | --- |
| Impact of caregiving | Impact on the mental and emotional well-being of the caregivers | *“We ask them to assist the patient in doing the physio; they just nod. They say It’s OK, sure, sure (small laugh). Their motivation is like low...probably because they are exhausted. They felt exhausted from taking care of the patient” (HCP 7 ; Physiotherapist)*  *“Ha, of course, I am stressed. With my child having a tantrum, PdPR (Home-based teaching and learning ) some more, because everything is on us, ha..cooking.. rushing..always rushing, keep looking at the clock. Always had to plan, oh after this, what should I do, then what….”(CG 4)* |
|  | Impact on the physical health of the caregivers | *“The father took care of the mom (patient) until he had a low blood pressure. When we called to see the patient, he said, I warded, because I have a low blood pressure, warded because of hypotension” (HCP 2 ; Domiciliary nurse)*  *The reason I stressed out was because I had to carry mom for bathing. Now, my brother-in-law helps a lot. Ha… I just give the shower; my brother-in-law will carry my mom. I had a hypertension because I kept carrying my mom. My blood pressure increased, haa……(CG 4)* |
|  | Impact on the social and professional life of the caregivers | *“Last time, we were in the process of preparing for a studio photo. All staffs is about 170.. To prepare, do the editing..so I have to bring the editing task back to home. Haa.. I settle the job while taking care of my mom…haa.. like that..have to…it s pretty disturbing when this thing happened, it s effecting my job at the office “ (CG 1)* |
| Needs of the Caregivers | Improvement of patient support and care | *“After that, in terms of assistance ..right..assistance…not that we expect to do this or that, the help that we meant like critical item .. critical item..haa '' wheelchair right ..sometimes even wheelchair they didn’t receive. So at home..patient is just lying down. Or the quaripod, they didn’t get the quadripod” (HCP 1; Rehabilitation Medicine Physician )*  *“Then of course, rapport. In this rehab, we must develop rapport with the therapist with the treating team because their impairment may take some time to recover, even as simple as LACI, face drooling at one side, but their self-esteem to start working, it s not just a week …no…it’s not…they are still afraid right, so I ll build rapport with them to empower for working, to involve in society, right from… they know to who they can talk to, they know if they encounter any issue, they have a team that they can refer to” (HCP 1; Rehabilitation Medicine Physician )* |
|  | Educational and Informational Support to Caregivers | *If for the children parts, maybe sometimes the children might not understand what stroke is, so they were, usually they'll complain, they will blame, uh, "will we see my father", or, "my father is so lazy, don't want to do all this". Yeah, yes. So the patient heard this, and then they will feel down, yeah, depressed.(HCP 6 ; Occupational Therapist)*  *“At that time, when my mom got the attack, we didn’t have any exposure to stroke. We blurred..so we don’t have exposure, so we don’t know what should we do, what the things we can do or cannot do, so at that time, we listen to lay people advise” (CG 1)* |
|  | Psychological support requirements and needs of Caregivers | *“Yes. Counselling for the caregiver. So that we can share our feeling, we can express our feeling, at least there is someone who listens to us. Hmm. Because I think, sometimes, people got depressed, and they cannot handle it and end up accidentally kill…., that kind…Because they felt, oh this patient is a burden to them. It s would be better if they were gone.haa..it’s like that. Who knows these things…” (CG 7)*  *“when their family members are affected by the stroke, OK, they they they they they become stressful, uhm, they sometimes they..they don't know, umm. What is best for the patient. Um, so, uh, yeah. I think we should support, their their their mental health because, uh, some may be a become frustrated with the family members who are affected by the stroke, and some may need financial support as well (HCP 10 ;Neurologist)* |
| Drivers for Caregiving (Internal and External) | Key Characteristics Of Caregiver | *“Ha, for example, like we ask, for example, physio to go teach, emm to avoid contracture, what so ever, but he didn’t do, like we teach again and again, he didn’t do, so like no cooperation, right” (HCP 2 ;Domiciliary nurse)*  *“The patient , the patient and caregiver, there are many categories of them, we can see from the beginning they come, when we explain about the treatment that they have to do at home, Home Programme, which supposedly to do at home, we can see some caregiver give their cooperation, some caregiver like, OK…it s OK,I ll do, some caregiver while we telling them, they busy talking to their phone, go there go here.so the explanation is not in full” (HCP 11; Occupational Therapist)* |
|  | Factors Within Stroke Survivor Themselves | *“If like to wear cloth, have to do it slowly, because she s like “It’s OK, I can wear it myself, even though its slow at the earlier, about 5 minutes to wear the cloth…a’aaa. But now she s able to do it in about 1 minute, nearly one minute to finish but she s do it herself” (CG 1)*  *“Hm, patient factor, I think mostly, I will relate to their motivation, and then their stress factor. Maybe they're, usually, they will have low motivation. From what I see, they will have low motivation in the beginning as they are quite anxious because they got stroke” (HCP 3; Occupational Therapist)*  *“He s lazy, he refuses to walk, He can, the therapy people said, he can. Can walk,uh walk on his own with the cane, the single stick cane. But he didn’t. Like he s not confident..ha..he s himself doesn’t feel confident to walk..to walk on his own because he is afraid he will fall (CG9)* |
|  | Family And Societal Support | *“From the financial part, sometimes we ask them to buy ..like ripple mattress..uh..the better one…em..then we ask to buy the walking frame, um..for ..uh ..to practice..Then we ask to buy..like patient just lying on the floor, so we ask to buy a proper bed..or we we can use any available bed in their home for instance..Ha.. so if the caregiver with poor financial, he has no money, he couldn’t buy all these things.” (HCP6 ; Occupational Therapist)*  *Most of it, most of them actually, their children taking care unless they really cannot, cannot support, that they can support them financially but physically they cannot, so they will reach a private nurse, or, or these, uh, Indonesian maid.(HCP 5; Physiotherapist)* |
|  | Restriction Due to Pandemic | *If I m not mistaken, most of the medical ward has been converted to covid ward, so most patients will be squeezed in one ward. So sometimes, when the ward is full, so like stroke patients, usually just been seen once, if stable, we will discharge them because there are not enough beds (HCP 3; Stroke ward nurse)* |
|  | Sub-Optimal Quality for Formal Stroke Care | *I think it is the same. I think, in in Malaysia the the post hospital care is not well developed in in, in, in, in our country. (HCP 10; Neurologist)*  *For the moment I feel, I do feel that I am trying my best to give the best but still, there are limitations in my part because we are not, we are just human, and we are not able to see all the patients, to all the patient needs. (HCP 6; Occupational Therapist )*  *“Meaning involvement, I meant from the wad, for me after the acute stage, I see a lacking. The was a lacking or like bombarded of information during the initial phase..what can do, what cannot do,all this like too much to caregiver” (HCP 1; Rehabilitation Medicine Physician)*  *For the bedridden patient, meaning like this domiciliary patient, they are a bit affected because we cannot go…. because lack of staff, sometimes our staff go to NCD team, our staff at PPV, so its really shortage staff for domi nowadays (HCP 2; Domiciliary nurse)* |
|  | Healthcare Provider Emotional Support | *“Usually, we will give emotional support. Tell them what should they do. To relative, we tell them ,they should be positive because if they give up, it’s will affect the patient. We will tell the caregiver to keep strong so that the patient will be strong as well” (HCP 8; Neurology clinic Nurse)* |
|  | Healthcare Provider Informational and Instrumental Support | *Then, I will urge them, I will explain to them, if you don't help, your father can't do this. So that's the, that's the outcome now. Now your father, I will, told the outcome for the, consequences for the, uh, caregiver, yeah, so for the next time, I will ask again (HCP 5; Physiotherapist)*  *“Uh, if the patients are a bed ridden, uh, immobilized, we, we also arranged for the patient to be visited by the staff of or from the nearest clinic. (HCP 10; Neurologist)* |
|  | Support For Essential | *If a bedridden patient, so far if from further away, we usually will ask to refer to the primary care centre, like the health clinic will see the patient and in case they need a consultation from the neurologist, they will call (HCP 8; Neurology clinic Nurse)* |
